# Experimental and Numerical Analysis of High-Temperature Superconducting Tapes Modified by Composite Thermal Stabilization Subjected to Thermomechanical Loading

**DOI:** 10.3390/ma14133579

**Published:** 2021-06-26

**Authors:** Eva Cuninková, Marcela Pekarčíková, Michal Skarba, Jozef Krajčovič, Matej Pašák

**Affiliations:** Faculty of Materials Science and Technology in Trnava, Slovak University of Technology in Bratislava, Jána Bottu 2781/25, 917-24 Trnava, Slovakia; marcela.pekarcikova@stuba.sk (M.P.); michal.skarba@stuba.sk (M.S.); jozef.krajcovic@stuba.sk (J.K.); matej.pasak@stuba.sk (M.P.)

**Keywords:** polymer-matrix composites (PMCs), thermomechanical properties, finite element analysis (FEA), high-temperature superconducting (HTS) tapes

## Abstract

The strain behavior of SiC/Stycast 2850 FT composites under thermomechanical loading using a finite element analysis (FEA) was studied. These composites can serve as thermal stabilizers of high-temperature superconducting (HTS) tapes during limitation event in resistive superconducting fault current limiter (R-SCFCL) applications. For this purpose, the thermomechanical properties of four composite systems with different filler content were studied experimentally. The FEA was calculated using an ANSYS software and it delivered useful information about the strain distribution in the composite coating, as well as in particular layers of the modified HTS tapes. The tapes were subjected to bending over a 25 cm core, cooled in a liquid nitrogen (LN2) bath, and finally, quenched from this temperature to various temperatures up to 150 °C for a very short time, simulating real limitation conditions. The outputs from simulations were also correlated with the experiments. The most promising of all investigated systems was SB11-SiC20 composite in form of 100 µm thick coating, withstanding a temperature change from LN2 up to 120 °C.

## 1. Introduction

Due to the immediate response of (RE)BCO (RE = rare-earth element, B = barium, C = copper, O = oxygen) HTS tapes, they have been the most suitable material for power applications such as R-SCFCL in the past decade [1]. A (RE)BCO HTS tape is a layered composite consisting of a metallic substrate, ceramic buffer, superconducting (RE)BCO and stabilizing Ag layers. However, the commercially available superconductors are mainly designed for cable applications [2,3,4], and they need to be further modified and adapted to the specific requirements of the R-SCFCL applications [5]. The working principle of R-SCFCL is based on a very fast switching from superconducting to non-superconducting (resistive) state. During a fault, the switch acts the moment the current exceeds the critical value (*I_c_*) of the superconductor. Consequently, the current passes through adjacent thin metallic layers (mostly from Ag) with following the limitation of the current due to the enhancement of the HTS tape resistivity [6]. As reported in [7,8], the (RE)BCO tapes may be damaged by a temperature rise during a quench. On one side, the thin metallic layer becomes highly resistive in accordance with needs, but on the other side, it generates a large amount of heat, which can rapidly (typically in 50 ms) overheat the tape to more than 250 °C, and thus can permanently damage the superconducting (RE)BCO layer of the HTS tape [9]. Because the heat conduction into the LN2 environment is very poor (due to the formation of a vapor film with thermally insulating properties [10,11]), it is important to increase the heat absorption capability of the tape itself, for instance by adding a layer or a coating possessing a high heat capacity, *c_p_*. Other important requirements for the stabilizing layer are its high electrical resistance and tailored coefficient of thermal expansion (CTE).

Nowadays, there are several concepts to develop new conductors suitable for the R-SCFCL technology: current flow diverter [12], conductor on flexible sapphire substrate [13], and metallic shunt with high electrical resistance [14]. The electrically conductive shunts, however, contribute to a reduction of the total electrical resistivity of the modified HTS tape and cause more heat to be generated at the same level of the electric field [15]. Therefore, an ideal thermal stabilization layer would be an electrical insulator. Our solution rests on the use of a polymeric material, which could be easily prepared in the form of a coating on the HTS tape with relatively low costs and without such thermal or mechanical side effects that would destroy the HTS ceramic layer during the fabrication process. The best candidates for this purpose are epoxy resins, which are widely used in cryogenic engineering areas as insulators, vacuum sealants, matrix materials for composites, etc. However, epoxy resins are generally brittle, in particular at cryogenic temperatures, and residual internal thermal stresses due to thermal cycling may be high enough to cause the epoxy resin to fracture. One of the main issues with the epoxy resins is, therefore, to mitigate the mismatch between the CTE of intended materials used for preparation of a stabilizing composite. The CTE of pure cured epoxy resins at room temperature (RT) ranges from 45 to 65 × 10^−6^ °C^−1^. This is too high in comparison with the Hastelloy C276 (12 × 10^−6^ °C^−1^), usually used as a substrate of the tape, and which is dominant, due to its major thickness, in determining of the HTS tape thermal expansion. In order to reduce the CTE of epoxy resin into the target range, various fillers with very low or even negative thermal expansion are added [16]. For example, fillers such as Al_2_O_3_, BN, SiC, SiO_2_, PbTiO_3_, or ZrW_2_O_8_ were used in order to control the thermophysical properties of the final composites [17,18,19,20,21]. The composite thermal stabilization, used in this study, is based on an epoxy resin matrix from Stycast 2850 FT (cured with Catalyst 11), which is specifically designed to resist thermal shocks, and has been used in a number of cryogenic applications, such as potting of superconducting magnet coils [22,23]. As a suitable filler, a SiC powder of various particle size and angular grains was chosen, based on our previous studies [24,25], where the thermophysical properties of several composite systems were investigated. The latter epoxy resin contains in as delivered state about 45 vol.% of an Al_2_O_3_ filler.

There are only a few studies on quenches of HTS tapes modified with high-*c_p_* materials in form of electrically non-conductive coating, due to the difficulty of temperature measurement in particular layers of the tape itself. The changes of the strain state at the layer interfaces induced by very fast heating-up and cooling-down event can influence the mechanical properties and it can also cause crack formation in the (RE)BCO layer, but strains are similarly difficult to measure. In such cases, a modeling tool is essential to improve our insight to the quench behavior of the HTS tapes with enhanced thermal stability by adding a material with high-*c_p_*. For the modeling, the thermophysical as well as the mechanical properties of all materials are essential for an accurate simulation. While the thermophysical properties of the Stycast-SiC composites were obtained by in-house measurements in our previous work [24], its mechanical properties were not available.

This work is focused on the investigation of thermomechanical strain state developed in particular layers of HTS tape modified by high-*c_p_* composite coating due to tape quench during limitation event, using a finite element method software (SW) ANSYS. The temperature dependence of the Young’s modulus (E), Poisson’s ratio (ν), and ultimate tensile strength (UTS) of the Stycast-SiC composites with different amount of the filler were measured as a function of a temperature, ranged from −120 °C to 100 °C in the frame of this study. The obtained mechanical and thermophysical properties, together with the available literature data, were the basis for the creation of several models describing the behavior of the samples in real experiments. The strain state of the particular models was compared in detail using a threshold criterion of strain, determined from the tensile tests.

## 2. Materials and Methods

### 2.1. Sample Preparation

**Figure 1:**
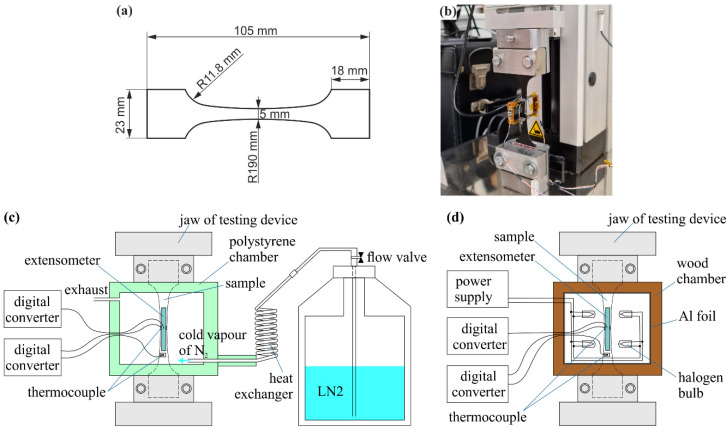
Tensile tests: (**a**) dimensions of a sample in mm; (**b**) tensile test setup with a sample measured at RT; (**c**) scheme of the experimental chamber for low-temperature testing; (**d**) high-temperature testing.

Four kinds of samples for tensile tests were prepared according to Table 1 showing the investigated systems. As a matrix, we used commercially available epoxy resin Stycast 2850FT cured by the hardener Catalyst 11 purchased from CMR-Direct (Somersham, UK). The epoxy resin Stycast 2850FT is usually called “Stycast Black”, so we will refer to it as “SB11”. In order to reduce the CTE of the epoxy resin, a SiC powder purchased from Merck (Bratislava, Slovakia) with typical angular shaped particles of various size in a range of 3‒100 µm was used as a filler. The choice of the filler is based on our previous experience. The composite SB11 with SiC filler possess the CTE (24 × 10^−6^ °C^−1^) in the temperature range from −140 °C to the temperature of glass transition, *T_g_*, near the CTE of silver. This is important in order to avoid the delamination of the composite layer. In terms of thermal conductivity, the mere addition of the SiC powder into the resin improved approximately two and half times the thermal conductivity of composites, compared to the pure SB without significant increase in the electrical conductivity. This composite can also withstand thermal cycling from LN2 to 150 °C (30 times) without a damaging effect [25]. The individual series of samples differed in the SiC content. A specified quantities of the epoxy resin and catalyst were mixed thoroughly before the SiC powder was added. The mix ratio (by weight) between epoxy and catalyst was 100:8, except the samples SB11-SiC20 and SB-SiC25 with a higher ratio of 100:8.5 and 100:10, respectively. Then, the mixture was mechanically stirred for 10 min at RT. During the mixing, the smaller particles of Al_2_O_3_ filler, which were present in the Stycast already in as delivered state, filled the gaps between the SiC plates, so the particles can touch each other and create quasi-conductive paths for heat flow. Once a homogeneous paste-like consistence of the mixed composite was achieved, it was embedded into a silicone mold, in order to produce the tensile test specimens with dimensions depicted in Figure 1a. The thickness of the samples was ~500 µm.

This is much more than the thickness of the coating used for HTS tape, which did not show any bubbles formation. In case of the samples for tensile test, an additional ultrasonic treatment for 2 min was applied in order to remove possible bubbles. Finally, the prepared samples were cured for 1 h at 125 °C, then removed of the mold, and subsequently post-cured for 4 h at 150 °C.

### 2.2. Methods

#### 2.2.1. Tensile Test

The samples were subjected to tensile loading using a universal testing machine UMZ-3K (MicroEpsilon, Bechyně, Czech Republic) at −120 °C, 22 °C (RT) and 100 °C with the test rate of 0.1 mm/min. For high- and low-temperature testing, two custom-made chambers were tailored to the working area of the tensile machine and to the dimensions of samples. The required temperature of samples was achieved with either N_2_ vapors blown into an extruded polystyrene chamber, or by a heat generation from light bulbs located near to the sample in the wood chamber insulated with aluminum foil (as shown in Figure 1c,d). The tests were performed at an approximately constant temperature (±3 °C), which was checked by a pair of thermocouples (type K) attached to the bottom and to the middle part of the samples. The stability of the high and low temperature was controlled by regulator of a power supply and by LN2 flow using a valve, respectively.

During the performed tensile test, the sample was under uniaxial tensile stress, as the shape of the sample is longitudinally symmetric. Figure 1b shows the experimental set-up of the experiment at RT. All measured signals were acquired with analogue-digital converters using data acquisition systems. The strain measurements were performed by means of an extensometer clamped by springs on the sample, with nominal gauge length of 20 mm. A double-extensometer configuration was used to avoid any bending effect related to the mismatch of the Young’s modulus data [25]. Both extensometers were wired together as a Wheatstone bridge type with two separate signal outputs. Before each tensile test, an exact gauge length was determined from the signal of each extensometer. The strain was calculated using the gauge lengths and the measured signals of the extensometers and then averaged. The reported results are an average of five measured samples.

The calibration of extensometers was verified using the digital micro-meter device INSIZE 3109-25B (Insize, Suzhou, China). The absolute accuracy was determined to be ±1 µm. To check the accuracy and the reproducibility of the complete measurement set-up, a hard copper sample was rigorously tested several times. In our experiments, Class 1 extensometers were used. Because of the relative bias error of ±1% or 3 μm lower limit, the absolute error in strain measurement increases as the strains become smaller. For instance, for a 25 mm gauge length and a 0.1% strain, the error in modulus can be as high as ±12%, and these are the typical uncertainties that can be attributed to the measurement of strain for a Class 1 extensometer [26].

#### 2.2.2. Thermophysical Properties

The density of the composites was determined by Archimedes method using Mettler Toledo precision laboratory scales (Mettler-Toledo, Bratislava, Slovak Republic). Demineralized water was used as an immersion liquid. The effect of the SiC particles on the density of the composites is presented in Table 1.

**Figure 2:**
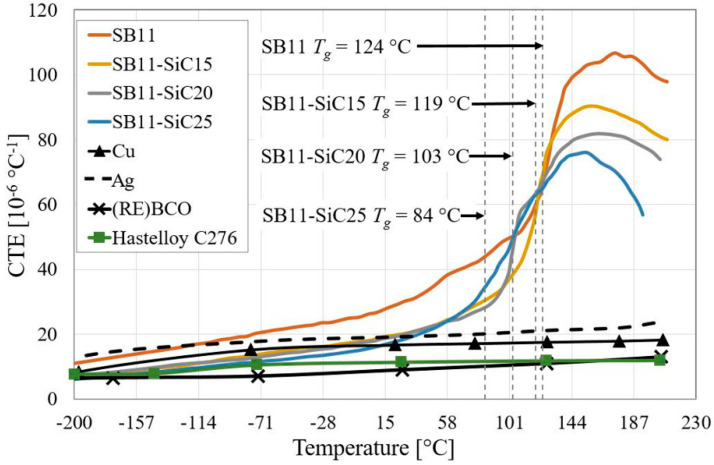
Temperature dependencies of CTE in all investigated composite systems. For comparison, the related data for Cu, Ag, Hastelloy C276 and (RE)BCO are plotted.

The CTE in the temperature range from −200 °C to 200 °C for all composite systems was determined on the base of our previous study [24] and is depicted in Figure 2. The CTE of SB11 was supplemented to −200 °C according to Swenson et al. [27]. The CTEs of other materials from the HTS tape in the same temperature range had to be inserted into the material data sources of the SW ANSYS. The CTE for Cu [28,29], Ag [30,31], and (RE)BCO [32,33] were taken from the literature, while the CTE data for Hastelloy C276 were measured in-house and correlated with results by Sugano et al. [34]. In the lower half of the investigated temperature range the CTE of all investigated composite systems increased almost linearly. After reaching the temperature of glass transition, the increasing trend continues, however, with a higher slope. These measurements showed that higher SiC content results in a mild shifting of *T_g_* to lower values (see Figure 2) [24].

Generally, the *T_g_* of cured epoxy depends on its chain flexibility, cross-linked structure, and the intermolecular hydrogen bonding interaction of the composites. With the addition of the SiC filler, the flexible segments would soften the cross-linked structure. Besides this, additionally added SiC particles increase the viscosity of the system, resulting in incomplete curing reactions. Therefore, the *T_g_* of the SiC/epoxy composites is lower than the *T_g_* of pure epoxy [35]. Additionally, shifting of the *T_g_* could be caused in particular due to the change in the mix ratio between used epoxy and catalyst. The use of higher ratio of catalyst in composites shifted its *T_g_* to lower temperatures.

#### 2.2.3. Mechanical Properties

**Figure 3:**
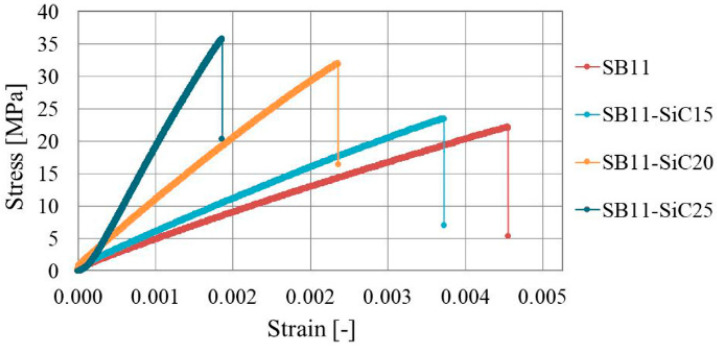
Stress-strain curves obtained from tensile tests for Stycast-SiC composites with different volume fraction of filler at RT.

Four representative results of tensile test for SB11, SB11-SiC15, SB11-SiC20, and SB11-SiC25 are plotted as engineering stress-strain curves in Figure 3. The tensile properties such as E, ν and UTS at −120 °C, RT and 100 °C are listed in Table 2.

**Figure 4:**
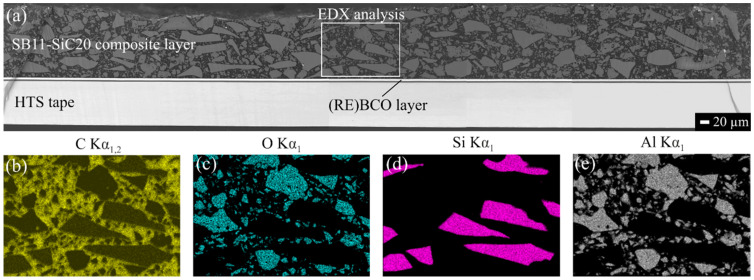
SEM image of the modified HTS tape in cross-section, with homogeneously dispersed filler in matrix of the composite coating (**a**) and its elemental distribution of: (**b**) C; (**c**) O; (**d**) Si and (**e**) Al confirmed by energy-dispersive X-ray (EDX) analysis.

The latter temperatures were chosen because of the experimental limits of the chambers during low- and high-temperature tensile tests. Based on our experimental results, the tensile properties were then extrapolated by linear fitting to −200 °C and 150 °C to meet design conditions in our FEA. As can be seen from Table 2, the UTS of the composites at RT increases linearly with the filler content. A similar trend was observable in *E*, which was expected, considering a homogeneously dispersed ceramic filler particles in epoxy matrix, investigated in cross-sectioned samples by scanning electron microscopy (SEM) and shown in Figure 4.

On the contrary, the ν was decreasing with increasing of the filler content. Since the added SiC particles are elastic, whereas the epoxy resin is viscoelastic, the ductility is thus reduced. Predictably, the SiC addition to the polymeric matrix diminishes not only the longitudinal strain (Figure 3), but also the transversal strain, resulting in a decreased ν for the composite material. Moreover, similar to the results in [36], the cryogenic strength is consistently higher than the RT strength for the same composition (Table 2).

When the temperature decreases from RT to −196 °C, chemical bonds and molecules will shrink and the binding forces between molecules will become stronger for the epoxy matrix. The thermal contraction of the epoxy matrix due to the temperature decrease causes the clamping force to SiC particles to increase at −196 °C thereto, leading to a stronger SiC/epoxy interfacial bonding [23]. Thus, a greater load will be needed to break the epoxy matrix at −196 °C, leading to a higher strength of the epoxy matrix at low temperatures. On one side, the increasing ratio of the SiC fillers content in the coating composite has been found to have a favorable effect on thermal expansion; on the other side, we noticed a decreasing effect on the elongation at break. In case of irregularly shaped filler particles, the strength of the polymer composites usually decreases as the partially separated microspaces between the SiC particles and the polymer obstruct the stress transfer from matrix polymer to the filler. Since the SiC particles are considered as solids, increasing filler content will lead to a reduction of the deformable polymer portion in the composite. This will obviously result in a reduction of the elongation at break, which was confirmed by results shown in Table 3. One can see in Table 3 that elongation at break of SB11-SiC25 at RT is much smaller than those of the other three samples. This behavior could be explained by a smaller CTE of SB11-SiC25 at RT in comparison with SB11-SiC20. Thus, the resulting elongation at the break of the composites could be partially affected by their CTE.

An almost monotonic decrease in elongation is notable as the SiC content in the composite increases to 25 vol.%. The cause of the decrease is the cross-linking between the neighboring ceramic particles with epoxy chain, which increases the rigidity and toughness of the composite system, but it decreases its ductility [37]. This sequence is disrupted only in the case of tensile testing at 100 °C. We suppose that the elongation values were influenced by *Tg*, which was for composites with 20 and 25 vol.% shifted close to or below the testing temperature.

For other materials within the HTS tape, the mechanical properties had to be defined in the models. The buffer stack was involved into the model with the same material properties as the (RE)BCO layer because of very similar material properties and negligible impact on the final results of the FEA. In order to input nonlinear material properties of Hastelloy C276 [38], Ag [39,40,41,42], (RE)BCO + buffer [43] and Cu [38] in temperature range from −200 °C to 200 °C, elasto-plastic model with bilinear isotropic hardening was used.

**Figure 5:**
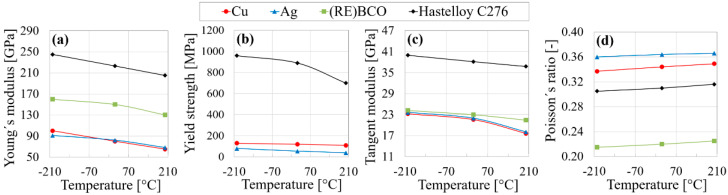
Tensile properties defined in material data source for numerical modeling: (**a**) Young’s modulus; (**b**) yield strength; (**c**) tangent modulus; (**d**) Poisson’s ratio.

Bilinear material curve was defined by Young’s modulus, E, yield strength, *σ_y_*, and tangent modulus, T, provided in Figure 5. Moreover, ν, as it is important in the determining of the effect of tensile and compressive stresses on the material, was specified in the material properties. It is difficult to measure the yield strength of the (RE)BCO material, as it tends to fracture before it enters the plastic deformation region, i.e., it is brittle. Therefore, the yield strength of (RE)BCO is missing in Figure 5b.

#### 2.2.4. Boundary Conditions for the FEA

The numerical calculations of strain distribution in such modified HTS tapes were made using a FE software ANSYS R18.1 Academic Workbench (ANSYS, Canonsburg, PA, USA). The models were created using 3D 8-node solid 185 structural solid elements under static structural simulation. Two kinds of models were built, differing in the composite coating thickness: (1) 100 µm, and (2) 200 µm. Other variables, included in the models, were the SiC content and the boundary conditions such as quenching temperature. Firstly, the created models of modified HTS tapes were bent on a copper anvil of 25 cm diameter. Second part of the simulation continued by cooling of the bent HTS tapes in LN2 and subsequently heated to a series of discrete quenching temperatures (75 °C, 100 °C, 120 °C, and 150 °C) for 50 ms, thus simulating the real limitation conditions in a R-SCFCL. The reason for bending of the modified HTS tapes in the FEA was to form a pancake coil, which is a typical shape for the HTS tapes in R-SCFCL. The quenching temperatures and time during the fault conditions were determined based on our previous study [44].

**Figure 6:**
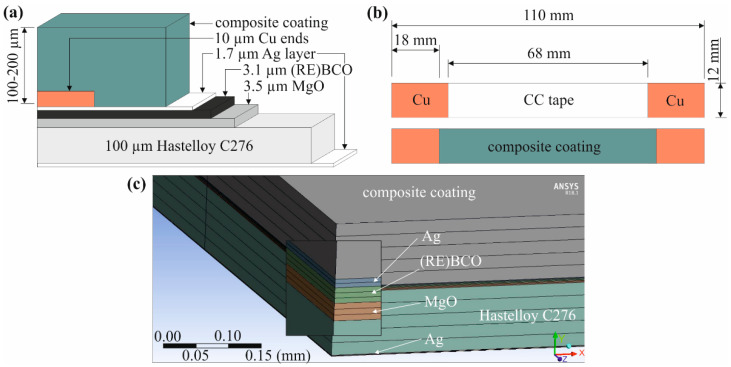
HTS tape sample coated with a composite layer: (**a**) configuration and thicknesses of the particular layers (not in scale); (**b**) dimensions of the sample for the model geometry; (**c**) FE mesh in the middle part of the model.

The geometry of the HTS tape with composite layer in the numerical model was based on real samples, described more precisely in [24]. In order to simulate the bending, Cu anvil with a diameter of 25 cm was created in the model. There, the multilayered HTS tapes were coated with up to 200 µm thick layer of the Stycast-SiC composite; the geometry and dimensions are shown in Figure 6.

Contacts between the layers of the HTS tape with composite layer were determined as bonded (no-slip) in all FEA. The contact between the surface of the bottom Ag layer of the HTS tape and the copper anvil was in frictional contact with the added coefficient of friction equal to 0.1. In the FEA, we assume that during the quenching the modified HTS tapes are completely surrounded by LN2.

Additionally, a model without the bending effect was calculated. This model was compared with experimental sample, which was not bent.

## 3. Results and Discussion

### 3.1. FEA of the Maximum Strains in the HTS Tapes Modified with the Composite Coating

Firstly, it would be interesting to look at the maximum equivalent strain in the whole material volume of particular HTS tape layers and to compare this state with some defined critical strain value. The threshold criterion of strain in the particular materials is based on a critical limit value, at which the degradation of the material occurs. According to our experimental results, the critical strain limits for the composite layers SB11, SB11-SiC15, SB11-SiC20, and SB11-SiC25 were 0.0181, 0.0120, 0.0165, and 0.0136, respectively (Table 3), which were determined by UTS at 100 °C. The threshold criterion of 0.0035 irreversible intrinsic strain in the (RE)BCO layer was defined as a critical limit value for the irreversible degradation of *I_c_*. This value was obtained by precise measurements in the work of Otten et al. [45]. Our calculated values of strain represent the absolute values because the residual strain from the production process (bending to the required radius) and the thermal strain (quenching during limitation) are taken into account.

**Figure 7:**
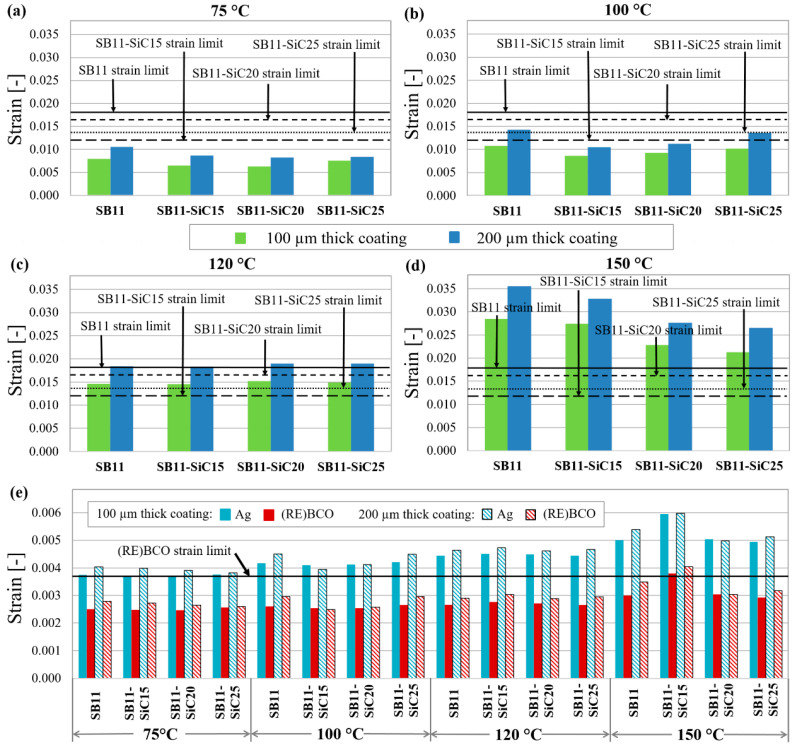
Maximum calculated equivalent strain of: (**a**–**d**) composite coating; (**e**) Ag layer and (RE)BCO layer in the samples with different filler content subjected to various quenching temperatures.

Figure 7 shows the calculated results of the maximum equivalent strain in the whole material volume of particular tape layers, with composite coatings of different SiC content subjected to four moderately different quenching temperatures (75 °C, 100 °C, 120 °C, 150 °C). As it can be seen from the Figure 7a–d, the higher the filler content, the lower the strain is induced in the composite coating after bending and quenching. This trend is slightly disrupted by the composite SB11-SiC25 (Figure 7a–c) and then at higher temperatures also by SB11-SiC20 (Figure 7b,c) with a small increase of the strain. We suppose that such behavior is strongly influenced by CTE, because the absolute calculated strain values copy the trend in CTE (see Table 4).

The strain is depending on the coating thickness, too. Higher strains are induced in thicker composite layer (200 µm in comparison with 100 µm), and this effect is clearly visible in every model at all quenching temperatures.

Nevertheless, all composite systems are under their strain limits at quenching temperature of 75 °C. During limitation event achieving 100 °C (Figure 7b), the lowest strain is generated in the SB11-SiC15 composite, and certain overrun of the strain limit can be seen for the 200 µm thick SB11-SiC25 coating. If we look at Figure 7c, it is evident that an overrun of the strain limit at 120 °C is reached in all 200 µm thick composites, regardless of the filler content. Thus for limiting in the case 120 °C is reached, the SB11 and the SB11-SiC20 coatings with 100 µm thickness could be suitable. All composites are, however, highly above their strain limits at the quenching temperature 150 °C (Figure 7d). With regard to the strain induced in the (RE)BCO layer, the limit 0.0035 was exceeded in models with SB11-SiC15 composite coating of both thicknesses at 150 °C, as obvious in Figure 7e. Since the strain limit in pure Ag is much higher [40] compared to the (RE)BCO and composite coating, the threshold criterion for this material was not designated and the strain distribution in Ag layer depicted in Figure 7e is shown only for comparison. Anyway, in a HTS tape modified with composite coatings, an attention to mechanical robustness must be focused on the composites, as they are the most exposed to mechanical strain during the limiting event as a result of the highest CTE among all materials used in our models.

### 3.2. FEA of Path Strain Distribution in the HTS Tapes Modified with the Composite Coating

**Figure 8:**
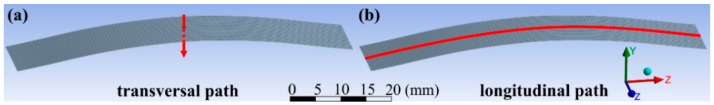
Schematic illustration of the methods for strain distribution analysis in the calculated models: (**a**) transversal path; (**b**) longitudinal path.

**Figure 9:**
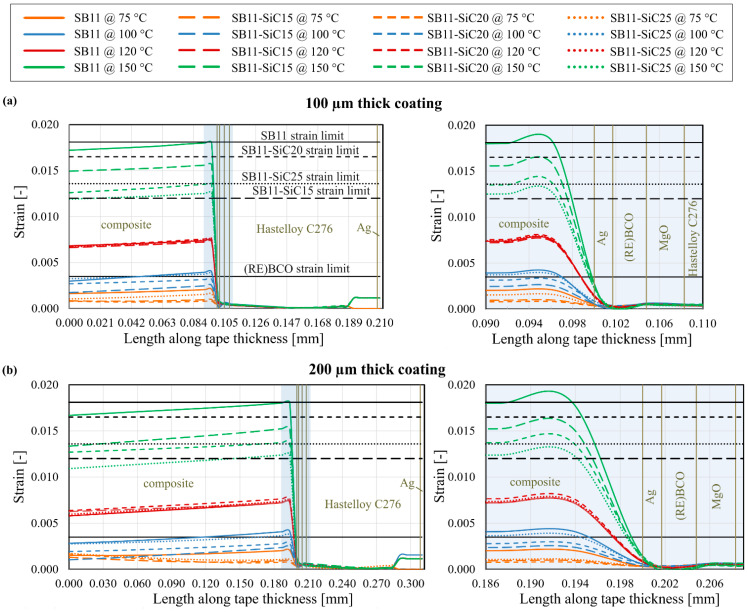
Equivalent strain distribution along the transversal path in the middle of the modified HTS tape at different quenching temperatures: (**a**) model with 100 µm and (**b**) 200 µm thick composite coating and different SiC content. For better clarity, the blue regions in plots on the left-hand side are magnified on the right hand side.

**Figure 10:**
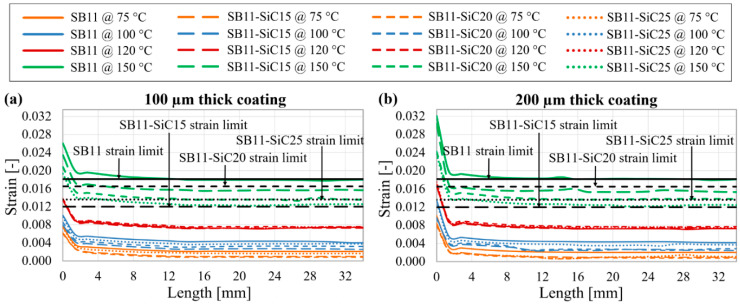
Equivalent strain of longitudinal path along the composite layer of tape: (**a**) model with 100 µm and (**b**) 200 µm thick composite coating, different SiC content and various quenching temperatures. For better clarity, only a half of the tape length is plotted; the strain distribution in the second half of the tape is approximately the mirror image of the opposite.

Another view of the strain distribution analysis is using a specific path or a surface plot of strain for the inspection in desired locations. We defined two kinds of paths. The first one represents the strain evolution through the thickness of the modified HTS tape (in the middle of the tape), depicted in Figure 8a as a transversal path. The second path represents the longitudinal path along the tape length, as can be seen in Figure 8b. The results of strain distribution along the defined paths are shown in Figure 9 and Figure 10.

Let us now discuss the strain distribution in the transversal direction. In the model with 100 µm thickness of the composite layer, the transversal strain distribution in the middle of tape proved that the critical strain limit was exceeded in the SB11 and the SB11-SiC15 composite materials exposed to 150 °C quenching temperature (see Figure 9a). The highest strain was located in the composite coating near to the interface with the Ag layer. The difference between the strain values of particular composites at 150 °C is significantly greater compared to those at 120 °C. We can find again a good correlation between the strain and the CTE values stated in Table 4, where the difference between the CTE values for the particular composite systems is also greater at 150 °C than at 120 °C. All lower quenching temperatures (75 °C, 100 °C, 120 °C) generate small enough strains in the middle part of the models, regardless of the SiC content.

A similar trend can be observed in the models with 200 µm composite thickness. Likewise, an overrun of the strain limits is visible only in the composites SB11 and SB11-SiC15 at 150 °C, as shown in Figure 9b. At quenching temperature 100 °C, the strain generated in SB11-SiC25 is interestingly higher than in the SB11-SiC20 coating at the same temperature, which could be caused by lower *Tg* = 84 °C of the SB11-SiC25 in comparison with SB11-SiC20 (103 °C). After reaching the *Tg*, CTE increases with a significantly higher slope, thus higher strain is generated in the SB11-SiC25. Pursuant to the strain limit of the (RE)BCO layer, the absolute strain is safely below the critical limit of 0.0035.

According to our previous observation, we would assume that from all the interfaces, the Ag/composite interface suffers the highest strain, because the high strain in the SB11-SiC layer is transmitted to this interface. Indeed, the maximal strain, for instance in 100 µm thick composite, is located outside the interface, in the composite material approximately 4 µm from the interface (see Figure 9a). This distance is 6 µm in the case of 200 µm thick coating, as seen in Figure 9b. Both distances are the same for all composites with different SiC content within the same thickness of composite. This indicates that region of maximal strain (in transversal strain direction) strongly depends on the composite thickness, as a result of shifting the neutral axis in the HTS tape with composite layer. Following the results of the Figure 9, a longitudinal path of strain in distance 4 µm and 6 µm from the Ag/composite interface for 100 µm and 200 µm thick coating, respectively, is plotted in Figure 10.

As evident from the latter figure, the highest strain is created at the edges of the modified HTS tape at each temperature. Moreover, the higher the quenching temperature, the higher strain gradient from the very edge to the more distant part of the tape can be observed. In the case of the 200 µm composite layer thickness, this effect is even more pronounced.

**Figure 11:**
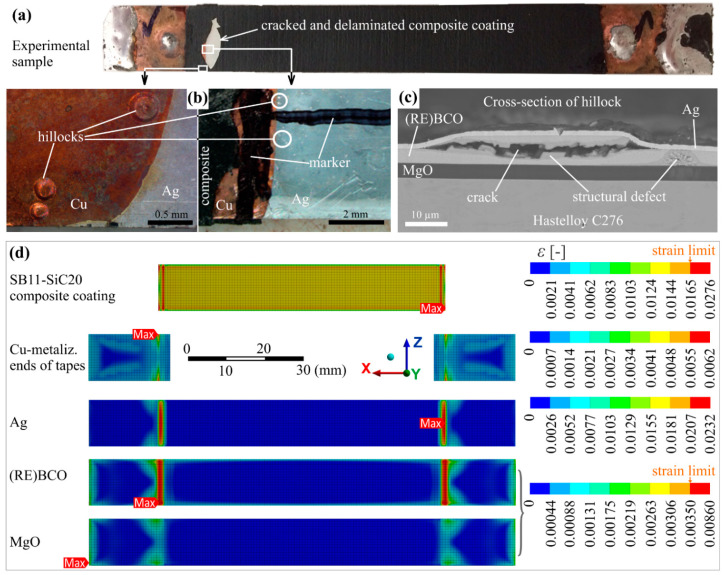
(**a**) Coated HTS tape with the SB11-SiC20 coating after 10 limitation cycles (E = 150 V/m, LN2, 50 ms DC pulse); (**b**) damage localized at the place of a cracked composite in form of hillocks visible in a light microscope; (**c**) SEM cross-section image of a hillock-like defect found in the place marked by circle in (**b**); (**d**) modeling results of a strain distribution in particular layers (top views) of the HTS tape modified with the SB11-SiC20 of 200 µm thickness and quenched at 150 °C.

The quenching temperature 75 °C is suitable for all composites, at 100 °C there is a slight overrun of the strain limit in 200 µm thick SB11-SiC25, the 120 °C introduces, in addition, limits in the coating thickness, and 150 °C is fully unacceptable because the ends of the coatings prove high strain level, increasing a risk of cracking or delamination. The latter effect could be observable even when the HTS tape is not subjected to a previous bending. Because our experiments on limitation were performed using unbent HTS tapes modified with the SB-SiC based composite, we also calculated the strain distribution in the models without the bending effect. The results are shown in Figure 11, and they can be correlated with the experimental results as follows.

In the limitation experiments at high electric fields of 150 V/m, described in more detail in [24], we observed that the 150–200 µm thick SB11-SiC composite layers coated on the HTS tape were cracked indeed at their ends after 10 limitation cycles, each lasting 50 ms (Figure 11a). The temperature of the tested samples was calculated from the measured electrical resistance, and it reached 150 °C as electric field 150 V/m was applied. The same samples limited before, using only 130 V/m, were heated to a temperature not higher than 60 °C and showed a good limiting performance without the *I_c_* degradation.

Of course, no *I_c_* degradation was recorded before the limitation, after coating the HTS tape with the composite. The good performance of the samples which underwent the electric fields up to 130 V/m during limitation corresponds well with the calculated strain development, which is below the strain limit for both the composite and the HTS layer at 75 °C and indicates no risk of cracking. Figure 11b shows hillocks formed in the HTS tape directly under the cracked composite layer.

**Figure 12:**
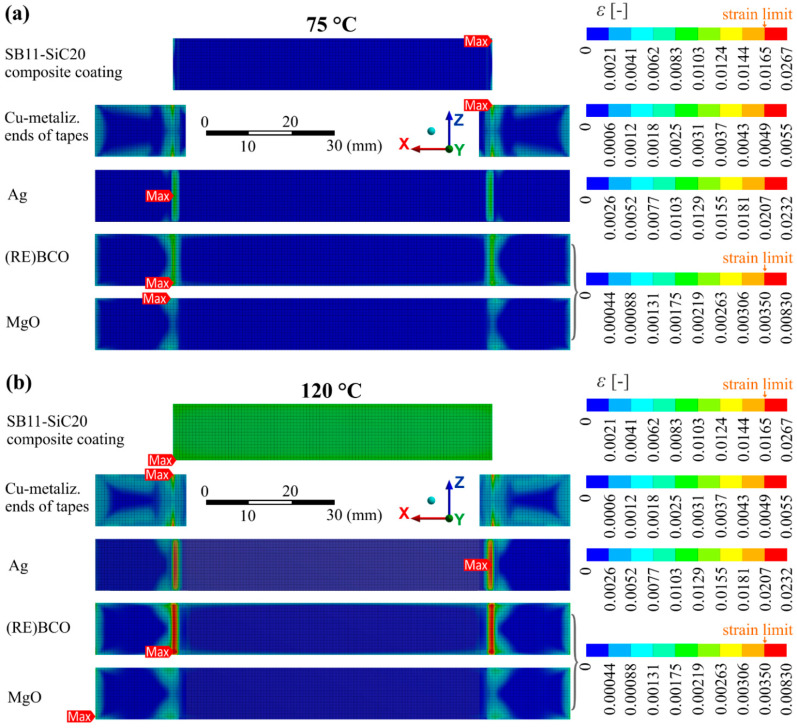
Modeling results of strain distribution in the particular layers (top views) of the HTS tape modified with SB11-SiC20 of 200 µm thickness and quenched at (**a**) 75 °C and (**b**) 120 °C.

An investigation of such hillock-like defects in cross-section by SEM showed structural defects in the superconductor, sometimes with a crack inside (Figure 11c). Their formation is not yet quite clear, but since they are observed indeed only in specific places of the tape (bellow ends of composite coating), they could by related to the maximal strain developed in the HTS layer, as indicates the calculated model of strain distribution in particular layers in Figure 11d at 150 °C. Figure 12 shows this situation at lower temperatures: at 75 °C, which can be defined as a safe state and at 120 °C, which is not critical for the composite coating but poses a danger for the superconductor. We suppose that places with high strain concentration would lightly deform the (RE)BCO crystal lattice and by this way they will contribute to the reduction of current transport capability with possible effect on hot spot formation in the HTS layer [46].

**Figure 13:**
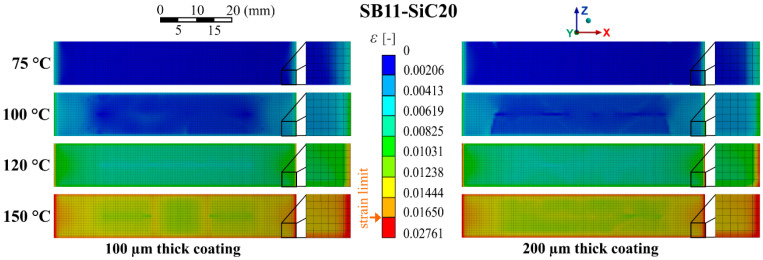
Distribution of the equivalent strain in the bottom composite coating surface (the surface in contact with Ag layer of the HTS tape) for the model with SB11-SiC20 coating of different thicknesses and quenching temperatures.

The best performing composite, based on the above mentioned evaluation of the maximal strain and strain in path distribution, seems to be the composite SB11-SiC20. Thus, more detailed information about the complex strain distribution in the bottom surface of the SB11-SiC20 composite layer (the surface in contact with the Ag layer of the HTS tape) is provided by contour plots of strain in Figure 13. Concerning the temperatures 75 °C and 100 °C, this composite is in a safe load conditions, for both thicknesses. The increasing concentration of strain at the ends and at the edges of the composite is evident in the case higher temperatures are applied. At the quenching temperature 120 °C, the strain limit is overrun for this type of composite (red ends). The thicker composite layer (200 µm) led to a further increase of the strain at the edges. A high overrun of the strain limit almost in the whole area of the interface is visible at quenching temperature 150 °C. Thus, this composite with up to a 200 µm thickness can be a good thermal stabilization of HTS tapes during limitation causing exposition to the higher temperatures (about 100 °C), as was proven by our experiments.

## 4. Conclusions

A mechanical strain distribution in the HTS tapes modified with a high-*c_p_* composite coating was studied using a 3D FEA. Several models were created using experimentally obtained data of the thermomechanical properties for four Stycast-based composite systems, differing in the SiC filler content. The models simulated the real limitation conditions. The main conclusions from the analysis are:

- The higher the filler content, the lower the maximal equivalent strain is induced in the whole material volume of the composite layer during the limitation. The trend is disrupted by the composite with the highest SiC content;
- The strain is distributed unevenly in the composite coating. The highest value is located at the ends of the coating in longitudinal direction. The transverse path showed that it is not exactly at the interface with the Ag layer as expected, but approximately 4–6 µm from this interface;
- Thicker coatings and higher quenching temperatures generate higher strain in all layers of the modified HTS tapes;
- Places with overlapping of more layer edges are the most sensitive to the high strain development. The superconducting layer must bear the strain in this location, which can cause its serious damage. The damaging of such places was also confirmed experimentally;
- The safest strain distribution was achieved with the SB11-SiC20 composite coating of 100 µm thickness and reaching the temperature lower than 120 °C. The calculated quenching temperature was 60 °C higher than the temperature measured in the experimental samples without the *I_c_* degradation. Herewith, the FEA recognized the possibility of using higher electric fields limits (>130 V/m).

## Figures and Tables

**Table 1:** Overview of the tested composite systems.

Sample	System Composition	Density [kg/m^3^]
SB11	Stycast 2850FT/catalyst 11	2271 ± 7
SB11-SiC15	Stycast 2850FT/catalyst 11 + 15 vol.% SiC	2390 ± 3
SB11-SiC20	Stycast 2850FT/catalyst 11 + 20 vol.% SiC	2691 ± 4
SB11-SiC25	Stycast 2850FT/catalyst 11 + 25 vol.% SiC	2992 ± 6

**Table 2:** Tensile properties of composites specified in FEA.

Composite	Experiment			Extrapolation		
	E [GPa]	ν [-]	UTS [MPa]	E [GPa]	ν [-]	UTS [MPa]
**−120 °C**				**−200 °C**		
SB11	21.32 ± 2.00	0.367 ± 0.010	44.00 ± 2.19	27.12	0.360	57.00
SB11-SiC15	24.11 ± 0.67	0.361 ± 0.010	54.84 ± 7.40	31.80	0.342	70.52
SB11-SiC20	26.42 ± 3.41	0.351 ± 0.005	59.47 ± 1.79	34.15	0.341	80.43
SB11-SiC25	29.80 ± 2.17	0.340 ± 0.020	65.50 ± 6.49	40.23	0.333	86.46
**RT**						
SB11	5.40 ± 1.72	0.369 ± 0.003	19.96 ± 3.50			
SB11-SiC15	8.66 ± 3.98	0.365 ± 0.006	24.74 ± 1.87			
SB11-SiC20	10.94 ± 7.35	0.360 ± 0.006	34.59 ± 2.72			
SB11-SiC25	15.61 ± 2.48	0.352 ± 0.004	35.93 ± 2.31			
**100 °C**				**150 °C**		
SB11	0.52 ± 0.27	0.382 ± 0.010	4.56 ± 0.08	0.15	0.384	1.50
SB11-SiC15	1.06 ± 0.05	0.377 ± 0.006	7.21 ± 0.81	0.40	0.382	3.00
SB11-SiC20	0.63 ± 0.31	0.371 ± 0.003	5.69 ± 0.86	0.20	0.376	2.00
SB11-SiC25	0.50 ± 0.30	0.363 ± 0.010	4.20 ± 0.72	0.10	0.367	1.45

**Table 3:** Elongation at break of composites determined from the experiments.

Sample	Elongation at Break [-]		
	−120 °C	RT	100 °C
SB11	0.00208 ± 0.00011	0.00504 ± 0.00197	0.01810 ± 0.00526
SB11-SiC15	0.00202 ± 0.00053	0.00417 ± 0.00132	0.01200 ± 0.00158
SB11-SiC20	0.00199 ± 0.00027	0.00326 ± 0.00172	0.01650 ± 0.00600
SB11-SiC25	0.00186 ± 0.00106	0.00181 ± 0.00102	0.01360 ± 0.00672

**Table 4:** CTE of the composite systems with various SiC content at different temperatures.

CTE [10^−6^/°C]	SB11	SB11-SiC15	SB11-SiC20	SB11-SiC25
22 °C	29.3	19.7	19.4	17.7
75 °C	41.6	28.1	26.9	29.2
100 °C	49.9	36.6	38.5	48.5
120 °C	61.0	62.0	66.5	64.9
150 °C	101.0	90.2	81.3	76.1

## Data Availability

Data are available on request to the corresponding author.
